# Establishment and characterization of stable red, far-red (fR) and near infra-red (NIR) transfected canine prostate cancer cell lines

**DOI:** 10.1186/s12935-020-01211-0

**Published:** 2020-04-29

**Authors:** Wen Liu, Sina Sender, Weibo Kong, Julia Beck, Anett Sekora, Kirsten Bornemann-Kolatzki, Ekkehart Schuetz, Christian Junghanss, Bertram Brenig, Ingo Nolte, Hugo Murua Escobar

**Affiliations:** 1 Division of Medicine, Clinic III, Hematology, Oncology and Palliative Medicine, Rostock University Medical Center, Ernst-Heydemann Str. 6, 18057 Rostock, Germany; 2 Small Animal Clinic, University of Veterinary Medicine Hannover, Hannover, Germany; 3 Chronix Biomedical, Göttingen, Germany; 4 Institute of Veterinary Medicine, University of Göttingen, Göttingen, Germany

**Keywords:** Prostate cancer, Canine, Cell line, Far-red, Near infra-red, Imaging

## Abstract

**Background:**

Canine prostate cancer represents a unique model for human prostate cancer. In vitro systems offer various possibilities but Xenograft in vivo imaging allows studying complex tasks as tumor progression and drug intervention longitudinal. Herein, we established three canine prostate carcinoma cell lines stably expressing fluorescent proteins allowing deep tissue in vivo imaging.

**Methods:**

Three canine prostate carcinoma (cPC) cell lines were stably transfected with fluorescent proteins in red, far-red and near infra-red spectrum, followed by G418 selection. Fluorescent protein expression was demonstrated by microscopy, flow cytometry and a NightOWL LB 983 in vivo imaging system. Cellular and molecular characteristics of the generated cell lines were compared to the parental cell line CT1258. Cell proliferation, metabolic activity and sphere formation capacity were analyzed. Stem cell marker expression was examined by qPCR and genomic copy number variation by genomic DNA whole genome sequencing.

**Results:**

Three stably fluorescent protein transfected cPC cell lines were established and characterized. Compared to the parental cell line, no significant difference in cell proliferation and metabolic activity were detected. Genomic copy number variation analyses and stem cell marker gene expression revealed in general no significant changes. However, the generated cell line CT1258-mKate2C showed uniquely no distal CFA16 deletion and an elevated metabolic activity. The introduced fluorescencent proteins allowed highly sensitive detection in an in vivo imaging system starting at cell numbers of 0.156 × 10^6^. Furthermore, we demonstrated a similar sphere formation capacity in the fluorescent cell lines. Interestingly, the clone selected CT1258-mKate2C, showed increased sphere formation ability.

**Discussion:**

Starting from a well characterized cPC cell line three novel fluorescent cell lines were established showing high cellular and molecular similarity to the parental cell line. The introduction of the fluorescent proteins did not alter the established cell lines significantly. The red fluorescence allows deep tissue imaging, which conventional GFP labeling is not able to realize.

**Conclusion:**

As no significant differences were detected between the established cell lines and the very well characterized parental CT1258 the new fluorescent cell lines allow deep tissue in vivo imaging for perspective in vivo evaluation of novel therapeutic regimens.

## Background

Canine prostate cancer (cPC) is a very aggressive disease, which is usually diagnosed at very late stages in veterinary patients [1]. In contrast to men, currently no screening markers are available allowing an early detection of the neoplasia. Additionally no gold standard therapeutic procedure has been established for the affected canine patients [2]. Thus, treatment options remain palliative with an expected remaining survival of weeks to months [3, 4]. Interestingly, canine prostate cancer has been lately focused as model for the human counterpart as cPC arises also spontaneously in presence of an active immune system sharing several histologic and biologic characteristics [5–7]. In contrast to men the incidence of cPC is rather low ranging between 0.2 and 0.6% [2, 8]. Concerning research the low incidence represents a major challenge as the availability of primary material is limited and thus large scale sample sets are rare. Consequently, cell lines are of major value in cPC research but are currently limited to a rather small number. Besides the herein used CT1258 cell line only a few further canine prostate cancer cell lines (as CPA-1, DPC-1, Ace-1, Leo, Probasco and CHP-1) have been reported [9–14].

In general, cell lines represent key tools in cancer research allowing the generation of neoplasias in animal models mimicking closely the initial tumors in vivo. Thereby, the combination of early stage in vitro settings and advanced stage in vivo models provides several possibilities to study therapeutic approaches and thus is prerequisite for rapid bench-to-bedside translation of anticancer therapies.

Advanced experimental approaches targeting complex tasks require the establishment of tumor specific in vivo animal models. Thereby, the characterization of early tumor development and the possibility to monitor tumor cell migration is of major interest for the evaluation of therapeutic agents. In previous studies we characterized the in vivo behavior of cPC derived cell line CT1258 in NOD/SCID mice and monitored tumor development in early stages by contrast enhanced 7T MRI [15, 16]. While MRI allowed longitudinal tumor development monitoring the method required cell labelling by supraparamagnetic nanoparticles or manganese [16]. However, these agents bare the disadvantage of no replicating during cell division, and the loss of signal intensity at certain time points.

The stable introduction of DNA coding for fluorescent proteins as eGFP and YFP offers an alternative allowing long term in vivo imaging without signal loss. While these early developed fluorescent marker proteins proved to be sufficient for most in vitro applications [17], in vivo imaging in deeper tissues requires fluorescent markers able to emit light in far-red or near infra-red wave length. Various systems have been established allowing substrate mediated (e.g. luciferase) as well as non-substrate mediated recombinant proteins (e.g. RFP) for deep tissue or whole body in vivo imaging. Both systems allow the detection of labelled cells in deeper tissues using whole body bioluminescence/biofluorescence-Imaging-Systems.

Fluorescence/luminescence-based monitoring of cancer development in vivo requires ideally a stable and long lasting expression of the fluorescent marker. Thus, a stable insertion of the acting recombinant proteins is a key for studies spanning observations in individual animals for several weeks. Commonly, lentiviral shuttle systems are used to stably transduce primary cells as well as cell lines for in vivo imaging applications [18]. While these systems deliver robust results showing high efficacy especially in difficult to transfect cells as primary cells and stem cells, the system also bares some disadvantages. Major disadvantages are e.g. laborious construction and purification of viral particles, handling with infectious agents, and lentiviral insertional mutagenesis. Akin to the viral transduction approaches, the stable integration of transfected plasmids bears the risk of insertional mutagenesis potentially affecting genomic stability and gene expression and thereby potentially altering the cellular behavior of the transfected cell lines. To characterize these potential changes the several characteristics of the newly established cell lines should be comparatively analyzed to the “original” lines. However, biofluorescent cell lines remain a key tool for the characterization of tumor development in vivo and thus for the evaluation of potential drug compounds.

Herein, we describe the establishment, characterization and validation of three canine prostate cell lines: CT1258-FusionRed, CT1258-mKate2C and CT1258-TurboFP650, that stably express different far-red and near infra-red fluorescent proteins. These cell lines provide valuable tools for study canine prostate cancer in vivo.

## Results

### Expression of FusionRed, mKate2 and TurboFP650 in transfected cell lines

**Fig. 1:**
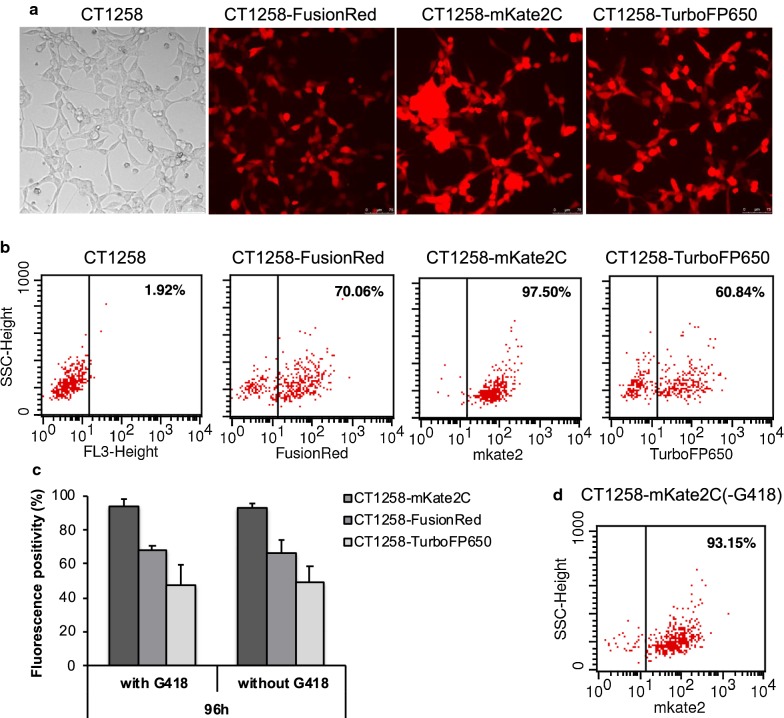
**a** mKate2C, FusionRed and TurboFP650 expression in stably transfected CT1258 cell lines. The magnification is 200× . **b** Example figures of flow cytometric analysis of fluorescent cell lines. Native CT1258 cells were used as negative control. Fluorescence was detected by FL-3 channel. **c** Fluorescence stability of transfected cell lines 96 h without G418 selection. Three independent experiments were performed for each assay. **d** Flow cytometric analysis of CT1258-mKate2C cells cultured 2 months in growth medium without G418

CT258 cells transfected with pFusionRed, pmKate2-C and pTurboFP650 expression vectors showed distinct red fluorescence 24 - 48 h post transfection, and achieved stable expression after approximate 30 days of cultivation and selection (Fig. 1a).

Flow cytometry revealed specific fluorescence in the fluorescent target wavelength compared to non-transfected CT1258 control cells. All transfected cell lines were measured three times after 2 months G418 selection using passage 1, 11 and 12. A mean percentage of 68.4% CT1258-FusionRed, 93.9% CT1258-mKate2C, and 47.56% CT1258-TurboFP650 positive cells were achieved under constant selection pressure (Fig. 1b). The single clone selected cell line, CT1258-mKate2C displayed the highest fluorescent positivity.

For cells grown in the presence of G418, the rates of fluorescent expressing cells were described above. In the absence of G418, mKate2 was 93.11% (CT1258-mKate2C), FusionRed was 66.2% (CT1258-FusionRed) and TurboFP650 was 48.9% (CT1258-TurboFP650). No differences were determined compared with the cells maintained under selective pressure (Fig. 1c). In addition, CT1258-mKate2C was cultured in medium in absence of G418 for 2 months, the amount of fluorescent cells remained 93.15% by flow cytometry analysis (Fig. 1d).

### Cell proliferation, metabolic activity and stability analyses

**Fig. 2:**
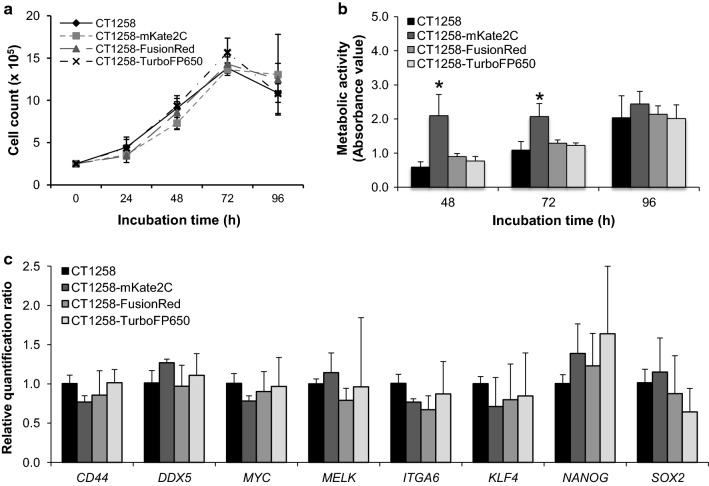
**a** Cell growth analyses by counting. **b** Metabolic activity analyses of native CT1258 and transfected cell lines. Significantly increased metabolic activity was determined in CT1258-mKate2C cells after 48 h and 72 h cultivation compared with native CT1258 cells. The Student’s *t*-test were performed and *p < 0.05 was assigned as significantly different. **c** Real-time quantitative RT-PCR was used to measure stem cell marker gene expression levels in native CT1258 and fluorescent cell lines. In CT1258-mKate2C, CT1258-FusionRed and CT1258-TurboFP650 cells, slightly increased or decreased expressions of stem cell marker gene were observed in comparison to native CT1258 cells. No significant difference was determined

The cell growth curves of native CT1258 and the three fluorescent cell lines were generated by four consecutive days of cell counting. CT1258 cells were used of passages 287, 290 and 294. Fluorescent cell lines were used from passage 4, 7 and 11. All fluorescent cell lines showed comparable growth characteristics to the untransfected CT1258 cell line (Fig. 2a). The respective initiating cell density was 2.5 × 10^5^ cells in 3.8 cm^2^. Untransfected CT1258 showed a population doubling time (PDT) of 29.2 h, CT1258-FusionRed of 28.6 h, CT1258-mKate2C of 29.4 and CT1258-TurboFP650 of 27.2 h. No significant difference was observed among the four cell lines.

The metabolic activity of each cell line is presented by the absorbance value at 450 nm wavelength. At 48 h and 72 h, CT1258-mKate2C cells displayed significantly higher metabolic activity compared with native CT1258 cells. The absorbance value was more than twice higher than native CT1258 cells at 48 h. CT1258-FusionRed and CT1258-TurboFP650 showed comparable metabolic activities to CT1258 cells increasing with cell number (Fig. 2b).

### Relative expression of stem cell marker genes in fluorescent cell lines

Relative real-time PCR expression analyses of the stem cell marker genes in generated fluorescent cell lines revealed no significant differences in all three fluorescent cell lines compared to the untransfected CT1258, although modest increase or decrease could be observed for distinct genes as for NANOG or SOX2 (Fig. 2c).

### The copy number variations of generated fluorescent cell lines

**Fig. 3:**
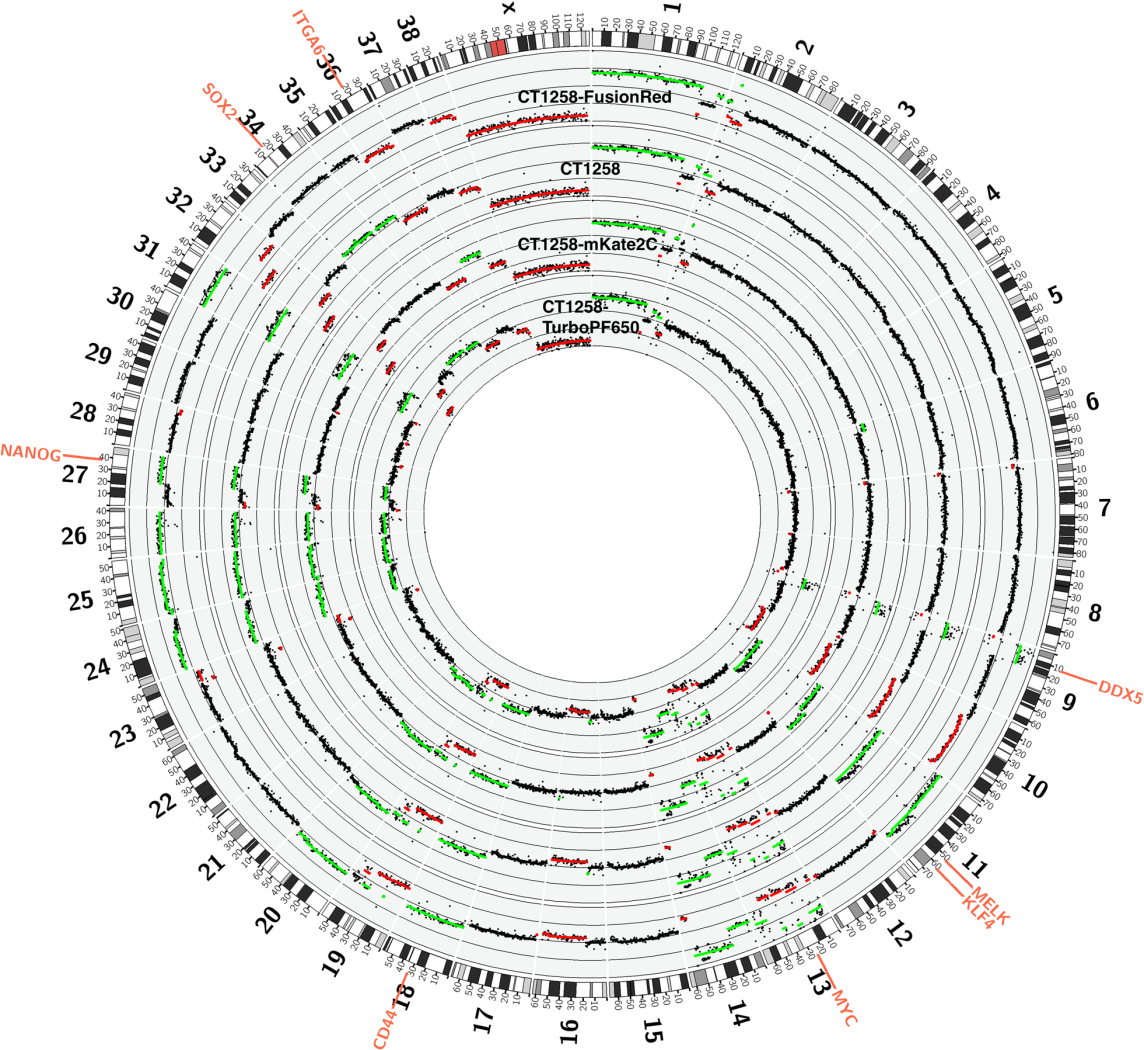
Circos plot of detected copy number variants in CT1258 and CT1258 fluorescent cell lines genomes. The canine chromosome ideograms are shown around the outer ring and distances in Mb. The next rings indicate successively the copy number information of CT1258-FusionRed, CT1258, CT1258-mKate2C and CT1258-TurboFP650. Each axis is scaled from 2 (outer border) to − 2 (inner border). Data were presented as log2-ratios. Copy number gain regions are highlighted in green and the loss regions are highlighted in red (thresholds: 0.2 and − 0.2)

The copy number changes refer to the canine genome (canFam3.1) and were identified by whole genome sequencing. The variations are presented in a Circos plot (Fig. 3). CT1258, CT1258-FusionRed, CT1258-mKate2C and CT1258-TurboFP650 showed in general comparable copy number variations. The respective variations are summarized in Table 1. Results are presented as log2 fold change values. The selected single clone CT1258-mKate2C cell line displayed the highest number of variations compared to CT1258, especially in the regions harboring the SOX2 and NANOG genes. Further CT1258-mKate2C is the only cell line which is not affected by a distal chromosomal deletion of CFA16 (chr16:18500001-59500001). By Ensemble, the region revealed approx. 270 genes located in the respective chromosomal area (Additional file 1). DAVID Functional Annotation tool analyses of the complete or partial gene list identified genes of lysosomal (four), insulin (three) and MAPK (three) pathway signaling to be harbored in the analyzed chromosomal region (Table 2).

**Table 1:** Copy number variations of stem cell marker genes (log2 fold change)

Gene	CT1258	CT1258-mkate2C	CT1258-FusionRed	CT1258-TurboFP650
*CD44*	0.3204	0.3398	0.2422	0.3476
*DDX5*	1.068	1.2312	1.1466	1.1015
*MYC*	1.7741	1.9331	1.7817	1.7519
*MELK*	0.4043	0.6948	0.3625	0.386
*ITAG6*	− 0.2812	− 0.6031	− 0.4024	− 0.3859
*KLF4*	0.4043	0.6948	0.3625	0.386
*NANOG*	0.1406	0.584	0.304	0.2508
*SOX2*	0.3109	− 0.0058	0.1956	0.3048

**Table 2:** Genes of KEGG pathway hits (DAVID functional annotation chart) in Chr.16 deleted region

Signaling pathway	p-value	Ensembl gene ID	Gene name
Lysosome	5.8E−2 (270 genes uploaded)	ENSCAFG00000007143	*N*-acylsphingosine amidohydrolase (acid ceramidase) 1
		ENSCAFG00000005586	Adaptor-related protein complex 3, mu 2 subunit
		ENSCAFG00000008381	Aspartylglucosaminidase
		ENSCAFG00000005378	Heparan-alpha-glucosaminide *N*-acetyltransferase
Insulin signaling pathway	8.4E−2 (51 genes uploaded)	ENSCAFG00000006154	Eukaryotic translation initiation factor 4E binding protein 1
		ENSCAFG00000005526	Inhibitor of kappa light polypeptide gene enhancer in B-cells, kinase beta
		ENSCAFG00000006673	Protein phosphatase 1, regulatory (inhibitor) subunit 3B
MAPK signaling pathway	8.1E−2 (51 genes uploaded)	ENSCAFG00000007750	Caspase 3, apoptosis-related cysteine peptidase
		ENSCAFG00000006845	Fibroblast growth factor 20
		ENSCAFG00000005970	Fibroblast growth factor receptor 1
		ENSCAFG00000005526	Inhibitor of kappa light polypeptide gene enhancer in B-cells, kinase beta

### In vitro imaging using NightOWL LB 983 in vivo imaging system

**Fig. 4:**
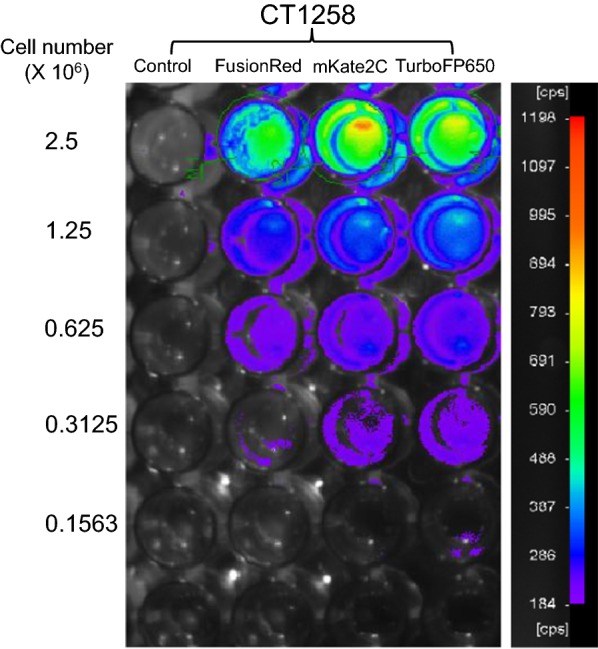
In vitro test using NightOWL LB 983 in vivo Imaging System. Images were taken using a filter with excitation of 590 nm and emission of 655 nm

Serial dilutions of the generated fluorescent cells were tested in vitro in a NightOWL LB 983 in vivo imaging system. The cell line CT1258-mKate2C displayed the strongest signal with an average counts per seconds (cps) of 226.13. Fluorescence of 0.156 × 10^6^ cells was detected for CT1258-TurboFP650 cells. At density of 0.3125 × 10^6^ cells, fluorescence signal can be detected for all cell lines. The signal of red protein FusionRed was relatively weak (Fig. 4).

### Sphere formation assay and the expression of the stem cell marker CD49f in CT1258-mKate2C

**Fig. 5:**
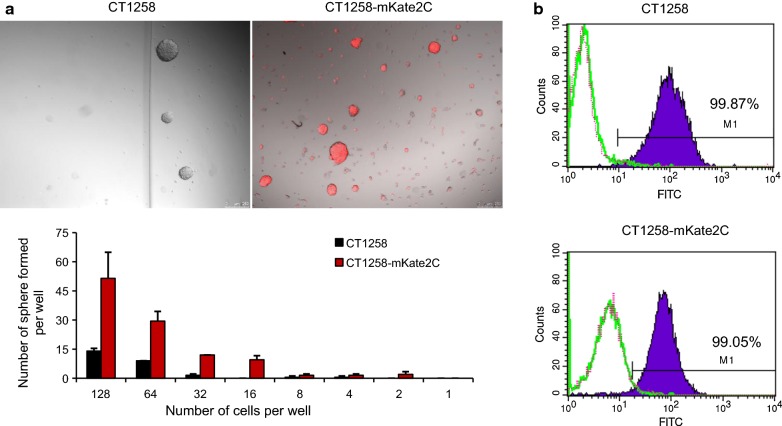
**a** CT1258 and CT1258-mKate2C cell lines were cultured in serum-free medium. Various sizes of spheres formed after 10 days in culture. The displayed picture shows the well with a number of 128 initially seeded cells. The formed spheres were counted under the microscope, 50 µm diameter or bigger were counted as a sphere. **b** The cell surface marker CD49f was analyzed by flow cytometry

In order to characterize if the clone selected cell line CT1258-mKate2C shows cancer stem-like cell behavior, sphere formation and CD49f expression were investigated. Under serum-free condition spheres with various sizes have formed from the cell lines CT1258 and CT1258-mKate2C. CT1258 derived spheres could be generated when more than 16 cells per well were initially seeded resulting in up to two spheres. At the density of 128 cells per well, the number of spheres ranged from 0 to 15. CT1258-mKate2C derived spheres were achievable already by single cell seeding in a well. Wells containing 128 cells resulted in 14 to 61 spheres (Fig. 5a). By flow cytometric analyses, similar expressions of CD49f were detected in CT1258 and CT1258-mKate2C cells. In both cell lines, more than 99% of cells were CD49f positive (Fig. 5b).

## Discussion

Dogs represent a valuable model for human prostate cancer as canine prostate cancer arises spontaneously sharing several similarities in presentation and biologic behavior to the human counterpart. In vivo models represent the key to understand the pathogenesis of prostate cancer and the development of novel therapeutic approaches. The cell line CT1258 established from a highly malignant adenocarcinoma with mesentery metastasis is one of the few available canine prostate cell lines showing highly tumorigenic behavior in vivo [15, 16]. In vitro systems offer several possibilities for basic drug evaluation but remain limited for the evaluation of complex interactions which must be analyzed in vivo. Thereby, fluorescent based reporter in vivo imaging offers the possibility to study the tumor progression as well as drug intervention longitudinal in animal models. However, precondition is the generation and detailed characterization of respective fluorescent cell lines.

In the current study, we established three cell lines stably expressing fluorescent proteins. CT1258-Fusionred and CT1258-TurboFP650 are two polyclonal transfected cell lines expressing FusionRed protein (red) and TurboFP650 protein (near infra-red), respectively. Under G418 selective condition, resistant cells outgrow non-resistant cells, resulting in a polyclonal population of stably expressing CT1258 cells. These populations maintained their heterogeneous character of the initial cell line CT1258.

CT1258-mKate2C represents a selected subline generated by limiting dilution assay. In CT1258-mKate2C, more than 90% cells are mKate2 positive and able to maintain a considerable fluorescent expression level after long-term cultivation even without selective antibiotic pressure. All cell lines have shown stable long time expression of their respective fluorescent protein. Further, fluorescent detection potential was verified by NightOWL LB 983 in vivo imaging system which is frequently used to monitor labelled cells in single animals over considerable time spans. The combination of the high NightOwl imaging sensitivity and the red/fR/NIR properties of the herein generated cell lines represent a valuable tool to study canine prostate cancer development and drug efficacy in vivo.

Stable undirected DNA integration can easily result in alteration of basic cell line characteristics. Comparative analyses of basic cell biologic characteristics as metabolic activity and doubling time revealed that the recombinant cell lines do not differ significantly from their parental cell line CT1258. While the major characteristics remained stable, an interesting observation could be made with clone selected cell line CT1258-mKate2C. During culture of the three fluorescent cell lines, cell cluster formation in CT1258-mKate2C was observed similar to a previous study in which sphere formation was achieved by serum-free CT1258 culture [19]. Matching these previous results an increased metabolic activity of CT1258-mKate2C was found consistent to our prior study characterizing the CT1258 generated spheres [19]. Therefore, the hypothesis arose that the CT1258-mKate2C cell line could be the result of a clone selected from a sphere-formation subpopulation. Previously, increased ITGA6 gene expression (a.k.a. CD49f) was detected in CT1258 generated spheres [19]. To clarify if CT1258-mKate2C is a result of a CT1258 sphere-formation subpopulation ITGA6 gene/surface marker CD49f (a.k.a. ITGA6) was comparatively analyzed in CT1258-mKate2C. The real-time PCR and flow cytometric results did not show increased expression of ITGA6 and CD49f in CT1258-mKate2C cells. However, under serum-free condition, CT1258-mKate2C cells revealed higher sphere formation ability compared to CT1258 cells. As the ability to form non-adherent spheres is one of the important phenotypic characteristics of cancer stem-like cells [20, 21], our results indicate that the CT1258-mKate2C cell line may have an enriched cancer stem-like cell population.

Further genomic profiling of the generated fluorescent cell lines showed in general no significant changes in the composition of copy numbers. However, again CT1258-mKate2C differed as this is the only compared cell line which does not show a distal CFA16 deletion. DAVID pathway analyses revealed three pathways which are modulated by twelve of the genes located in the preserved CFA16 part.

Matching the general genomic CNV data the analyzed stem cell marker expression also showed no significant variation. Consequently key aspects of the generated cell lines remained comparable to the parental line allowing transferability of earlier generated functional results achieved with CT1258 [16, 17].

## Conclusion

In general, in this study we established and characterized stably transfected canine prostate carcinoma cancer cell lines in vitro for deep tissue in vivo imaging. The generated CT1258-FusionRed, clone selected CT1258-mKate2C and CT1258-TurboFP650 cell lines kept their parental cell line characteristics and showed stably strong fluorescent protein expression. As the cell lines express their respective marker in red, far-red and near infra-red spectrum the generated cell lines provided a valuable option for deep tissue in vivo imaging and with that the possibility for later in vivo evaluation of novel therapeutic regimens.

## Materials and methods

### CT1258 cell line

The canine prostate adenocarcinoma cell line CT1258 established and characterized previously by us was used as parental cell line [15, 22]. Cells were regularly tested for mycoplasma contamination.

### Expression vectors and plasmids preparation

Three mammalian vectors pFusionRed-C, pmKate2-C and pTurboFP650-C (Evrogen, Moscow, Russia) were used for transfection. The vectors respectively encode red fluorescent protein FusionRed, far-red fluorescent protein mKate2 and near infra-red fluorescent protein TurboFP650. All three vector backbones contain a neomycin resistance gene (Neo^r^) allowing selection of stably transfected cells using Geneticin^®^ Selective Antibiotic (G418) (Life Technologies, Darmstadt, Germany). The vectors were transformed into thermocompetent E. coli DH5α cells according standard procedures. Plasmid DNA was extracted from isolated and expanded culture bacteria using NucleoBond^®^ PC 500 plasmid DNA purification Kit (MACHEREY–NAGEL GmbH, Düren, Germany).

### Transfection of CT1258 cells

The CT1258 cells were plated 5 × 10^5^ cells per well in 6-well plate 24 h before transfection allowing the cell to attach and rest. Transfection reactions were performed using X-tremeGENE HP DNA Transfection Reagent (Roche, Mannheim, Germany) according to the manufacturer’s protocol. Briefly, X-tremeGENE HP DNA Transfection reagent, plasmid DNA and transfection diluents Opti-MEM Reduced Serum Media (Life Technologies) were allowed to equilibrate to room temperature (RT). For each transfection, 2 µg plasmid DNA was diluted in Opti-MEM media to a final volume 200 µl. Following, in each sample 6 µl transfection reagent were added into the medium containing the plasmid DNA and mixed gently. The transfection complex was incubated 15 min at RT. After adding the transfection complex to the respective cells, the respective plates were incubated in a humidified 5% CO_2_ incubator for 48 h at 37 °C. The same number of cells was respectively seeded in three additional wells (without DNA, without transfection reagent, and only cells) serving as untransfected control. The expression of the fluorescent protein was verified using a Leica DMI 4000B fluorescence microscope (Leica Microsystems GmbH, Wetzlar Germany).

### G418 selection and expansion of stably transfected CT1258 cells

The G418 kill curve generation of CT1258 was performed previously by us [17]. Post transfection (48 h) G418 was applied at a dose of 600 µg/ml to each transfected well. As a control to assess the antibiotic response, the same dose of G418 was applied to the untransfected cell control well. The cells were examined daily and medium changed every 2 days. The cells which integrated the transfected plasmid are supposed to survive the G418 selection while cells without transfected plasmid integration will be eliminated. G418 selection was carried out until all untransfected control cells were eliminated. Following, the respective remaining vital cells were expanded in T25 cell culture flasks. After 2 months high dose G418 selection, the concentration was reduced to 300 µg/ml for further cultivation of the generated cell lines.

### Fluorescence expression analysis by microscope and flow cytometry

During G418 selection, the three transfected cell lines were controlled by fluorescence microscopy weekly. The respective amount of fluorescent transfected cells were analyzed by flow cytometry. Therefore, the cells were trypsinized and dissociated into single cell suspension, adjusted 1 × 10^6^ cells in 500 µl phosphate buffered saline (PBS), and examined using the FL-3 channel in a FACS Calibur (BD Biosciences, Heidelberg, Germany). Data analyses were performed with Cell Quest soft-ware (BD Biosciences, Heidelberg, Germany).

To further characterize the stability of the fluorescent cell lines, the respective fluorescent cell rates were measured by flow cytometry after 96 h cultivation without G418.

### Identification and cultivation of CT1258-mKate2 clone by limited dilution

In order to preserve the heterogeneous character of the cell line CT1258, the transfected cell lines are preferable to keep the original CT1258 polyclonal character. However, despite the G418 selection a population of untransfected cells remained in the cell lines. We identified and selected single clone from CT1258-mKate2 transfection by limited dilution and cultivated them separately further. This cell line was named CT1258-mKate2C.

The selection was done as follows: CT1258-mKate2 cells were diluted to a density of 10 cells/ml by selection medium and seeded 100 µl per well in 96-well plate. After four days, the number of colonies was assessed in the wells. Only wells with one colony per well were marked for further analyses. Fluorescence expression was identified by fluorescence microscopy of the remaining colonies, two positive colonies were selected for further expansion. Fluorescence was further verified by flow cytometry after three passages, the clones showing highest expression were kept in culture and frozen for long-term storage.

### Analyses of cell proliferation and metabolic activity

The CT1258, CT1258-FusionRed, CT1258-mKate2C and CT1258-TurboFP650 cells were seeded 2.5 × 10^5^ cells per well in four 12-well plates adding 2 ml of medium without G418. The number of viable cells was determined at 24, 48, 72 and 96 h by trypan blue staining using a conventional cell count chamber. Population doubling time (PDT) was calculated by the formula PDT = 1/[3.32(logNH-logNI)/(t2 − t1)] (t1 = time in hours when cells were seeded; t2 = time in hours when cells were harvested; NI = cell count at time cells were seeded; NH = cell count at time cells were harvested). Herein, cells were harvested after 72 h to calculate the PDT.

Metabolic activity measurements were performed by WST-1 assay. In a 96-well plate, 1.5 × 10^4^ cells per well were plated in triplicate in 150 μl medium without G418. Metabolic activity was analyzed after 48, 72 and 96 h using tetrazolium compound WST-1 reagent (Roche, Mannheim, Germany). Absorbance at 450 nm and the reference wavelength at 750 nm were determined by GloMax^®^-Multi Detection System (Promega GmbH, Mannheim, Germany). All experiments were repeated three times independently.

### Stem cell marker gene expression analyses

As stem cell marker expression is crucial in cancer cell lines a distinct marker panel covering the canine genes CD44, CD133, c-KIT, CD34, ITGA6, MYC, NANOG, DDX5, KLF4, SOX2, MELK and OCT4 (assay details see previous reports [19, 23]) was analyzed comparatively by relative quantitative real-time PCR (qPCR) to evaluate stable transfection induced expression changes among CT1258 and fluorescent cell lines CT1258-FusionRed, CT1258-mKate2C and CT1258-TurboFP650.

Total RNA was extracted from CT1258 and fluorescent cells using RNeasy mini Kit (Qiagen, Hilden, Germany). cDNA synthesis was carried out using 500 ng of total RNA in 20 µl according to the manufacturer’s protocol for the QuantiTect Reverse Transcription Kit (Qiagen, Hilden, Germany). The qPCR reactions were performed using the ViiA™ 7 Real-Time PCR System (Life Technologies) and QuantiTect SYBR green qPCR Kit (Qiagen). ß-actin (ACTB) and Glyceraldehyde-3-Phosphate Dehydrogenase (GAPDH) were used as endogenous control. The qPCR results were analyzed using the delta delta CT (ΔΔCT) method relative to CT1258 cells. For each cell line, three samples of different passages were used. All samples were analyzed in triplicates including non-template and non-reverse transcriptase controls for each reaction.

### Genomic DNA extraction and sequencing for genomic copy number variation (CNV) analyses

As Genomic DNA was extracted from cultured CT1258 and CT1258 fluorescent cell lines using the NucleoSpin^®^ Tissue Kit (MACHEREY–NAGEL GmbH, Düren, Germany) following the manufacturer’s instructions.

200 ng genomic DNA was ultrasonically sheared and sequencing libraries were prepared using the NEBNext Ultra DNA Library Prep Kit (New England Biolabs, Frankfurt am Main, Germany) according to manufacturer’s instructions. Shallow shotgun sequencing (single read, 150 bp) was conducted on a NextSeq 500 (Illumina, San Diego, CA, United States) yielding an average of 14 M reads (SD: 6 M). Reads were aligned to the canine reference genome (version: Broad canFam3.1). After duplicate removal using Picard Tools (http://picard.sourceforge.net) the sequence reads in 500 kbp bins were counted, the numbers were corrected for mappability and GC content and log2-transformed copy-number ratios were called using the QDNAseq R-package [24]. Obtained copy-number data were smoothed by applying circular binary segmentation using the DNAcopy R-package [25].

### DAVID pathway analyses

The genes in the chromosomal deletion region of CFA16 (chr16:18500001-59500001) were identified based on Ensembl database. The complete gene list was uploaded to the DAVID Functional Annotation tool (https://david.ncifcrf.gov/tools.jsp) for the analysis of associated pathways. Furthermore, the gene list was converted by DAVID Gene ID Conversion tool and partial genes were submitted to DAVID Functional Annotation tool for pathway analysis.

### In vitro imaging using NightOWL LB 983 in vivo imaging system

Viable cells were counted and plated at a density of 2.5 x 10^6^ per well in a 96-well plate and followed by a 1:2 serial dilution until 0.156 x 10^6^ cells per well. The 96 well plate was placed in the NightOWL LB 983 imaging system (Berthold Technologies, Bad Wildbad, Germany). Photo was taken using a filter with excitation of 590 nm and emission of 655 nm.

### Limited dilution sphere formation assay and flow cytometric analysis of CD49f

The capacity of sphere formation of CT1258 and CT1258-mKate2C cell lines were examined additionally. The cells were prepared as a single-cell suspension in serum-free medium. The medium consisted of DMEM/F12 (Biochrom, Berlin, Germany), supplemented with 5 µg/ml Insulin (Sigma-Aldrich, Seelze, Germany), 20 ng/ml human epidermal growth factor (EGF) (Biochrom), 20 ng/ml human basic fibroblast growth factor (bFGF) (Life Technologies), and 2% B27 (Life Technologies). Different cell numbers varying from 128 to 1 cell/per well were seeded in 96-well plates with duplicates. The number of generated spheres was counted after 10 days cultivation. The medium was changed every 3 days. The assay was performed three times independently.

CD49f (a.k.a. ITGA6) expression on the cell surface was detected by flow cytometry. CT1258 and CT1258-mKate2C cells were trypsinized, washed with PBS twice and then resuspended in PBS. For each measurement, 1 × 10^6^ cells were placed in 100 µl PBS with 1% BSA in a flow tube. Cells were incubated with 1 μg rat anti-human CD49f (clone GoH3, BD Bioscience, Heidelberg, Germany) at 4 °C for 30 min in the 100 µl PBS [26]. After washing twice with cold PBS, cells were incubated with 1 μg rabbit anti-rat FITC (STAR17B; AbD Serotec, Puchheim, Germany) antibody for 30 min at 4 °C in the dark. After incubation, the labeled cells were washed with PBS, resuspended in 400 μl PBS and analyzed using a FACSCalibur flow cytometer (BD Bioscience). Analysis was done using the CellQuest (BD Bioscience) software. Rat IgG2aκ purified (BD Bioscience) was used as isotype control. All measurements were carried out twice.

### Statistical analysis

The Significant differences were calculated using Student’s *t* test, where a *p*-value of less than 0.05 was considered to be statistically significant.

## Supplementary information

**Additional file 1.** Genes located in the chromosomal area chr16:18500001-59500001.

## Data Availability

All data generated or analyzed during this study are included in this published article and its additional files.

## Abbreviations

cPC: Canine prostate cancer
eGFP: Enhanced green fluorescent protein
fR: Far-red
G418: Geneticin
Neo^r^: Neomycin resistence gene
NIR: Near infra-red
PDT: Population doubling time
RFP: Red fluorescent protein
YFP: Yellow fluorescent protein
